# The 30-year cardiovascular risk profile of South Africans with diagnosed diabetes, undiagnosed diabetes, pre-diabetes or normoglycaemia: the Bellville, South Africa pilot study

**DOI:** 10.5830/CVJA-2010-087

**Published:** 2012-02

**Authors:** Tandi E Matsha, Mogamat S Hassan, Martin Kidd, Rajiv T Erasmus

**Affiliations:** Department of Bio-Medical Sciences, Faculty of Health and Wellness Science, Cape Peninsula University of Technology, Cape Town, South Africa; Department of Nursing and Radiography, Faculty of Health and Wellness Science, Cape Peninsula University of Technology, Cape Town, South Africa; Centre For Statistical Consultation, University Of Stellenbosch, Cape Town, South Africa; Division Of Chemical Pathology, Faculty Of Health Sciences, University Of Stellenbosch, Cape Town, South Africa

**Keywords:** cardiovascular diseases, diabetes mellitus, epidemiology, obesity, South Africa

## Abstract

**Abstract:**

The aim of this pilot study was to assess the 30-year risk for cardiovascular disease (CVD) in the South Africa population of mixed-ancestry in individuals with non-diabetic hyperglycaemia, and undiagnosed and self-reported diabetes. Participants were drawn from an urban community of the Bellville South suburb of Cape Town. In total, 583 subjects without a history of CVD were eligible for lifetime CVD risk estimation. Gender-specific prediction for CVD risk was calculated using the 30-year CVD interactive risk calculator. High CVD risk (> 20%) was evident in normoglycaemic and younger subjects (under 35 years). The significant predictors of CVD were sibling history of diabetes, and triglyceride, low-density lipoprotein cholesterol and glycated haemoglobin levels (*p* < 0.001). The high lifetime risk in normoglycaemic and younger subjects may be considered a warning that CVD might take on epidemic proportions in the near future in this country. We recommend the inclusion of education on CVD in school and university curricula.

## Abstract

Urbanisation, and demographic and epidemiological transitions have rendered diabetes one of the major non-communicable diseases in South Africa. Studies carried out in South Africa have shown marked geographical and ethnic variations in the prevalence of diabetes.^1^-^3^ The mixed-ancestry population of South Africa has the second highest prevalence of diabetes, preceded by that of the Indians.^1^, ^3^ Diabetes mellitus (DM), particularly type 2 DM is highly associated with cardiovascular disease (CVD), and mortality from CVD is two- to four-fold higher in those with the disease.^4^ In South Africa, CVD is the second leading cause of death after HIV/AIDS,^5^ and the South African National Department of Health has identified diabetes as a major risk factor.^6^

The relationship between dysglycaemia and CVD is linear and sometimes starts below the diagnostic levels of diabetes.^7^, ^8^ Of the two pre-diabetic states, impaired fasting glucose (IFG) and impaired glucose tolerance (IGT), the relationship between IGT and CVD is well documented, however that of IFG and CVD is controversial.^9^, ^10^ Fasting glucose levels compared to postprandial glucose concentration were found not to be the strongest determinants of intima–media thickness (IMT) or death associated with hyperglycaemia,^7^, ^9^ however, the DECODE study group^7^ did find an increased risk of mortality for individuals with IFG compared to those with normoglycaemia. In developing countries, diabetes is often undiagnosed and the risk of CVD may therefore far exceed that of known cases due to the unmanaged glycaemic state.

Various mathematical equations that incorporate the major risk factors (age, gender, high blood pressure, smoking, dyslipidaemia and diabetes) have been developed for the assessment of CVD risk over a 10-year period in general populations.^11^-^14^ The performance of the two frequently used models, the Framingham Heart Study^13^ and the UK Prospective Diabetes Study risk engine (UKPDS), version 3,^14^ have been evaluated in individuals with diabetes, hyperglycaemia and normoglycaemia.^15^ The Framingham Heart Study performed better at classifying subjects with a net gain in correct classification of –14 and –12.4% for non-diabetic hyperglycaemia and normoglycaemia, respectively.^15^ However, the 10-year time frame of these models has been criticised because an individual’s lifetime risk may be high while the 10-year risk prediction may be low, therefore delaying efforts to modify that risk.

Recently, an algorithm that allows for 30-year risk assessment for individuals with any combination of risk factors has been developed.^16^ While the 30-year risk equation provides a lifetime risk level, it is thought that this method oversimplifies the risk and may also lead to overuse of medication. Other limitations of this method include patient-specific issues, such as socio-economic status and ethnicity.^17^

In Africa, large prospective studies to recalibrate these CVD equations to correct for differences in baseline survival between ethnic groups^18^ are restricted by poor documentation of causes of death as well as migration of individuals. The South African adult population has high levels of CVD risk factors, and the INTERHEART Study^19^ showed that several of the risk factors for myocardial infarction operate similarly in different ethnic groups and geographical locations worldwide.^20^

The aim of this pilot study was to assess the lifetime CVD risk in the mixed-ancestry population of South Africa in individuals with non-diabetic hyperglycaemia, undiagnosed diabetes, known diabetes and normoglycaemia, using the recently developed 30-year CVD interactive risk calculator.^16^

## Methods

The Faculty of Health and Wellness Sciences Ethics Committee of the Cape Peninsula University of Technology approved the study, which was conducted according to the code of ethics of the World Medical Association (Declaration of Helsinki). All participants signed written informed consent after all the procedures had been fully explained in the language of their choice. In addition, permission was also sought from other relevant authorities such as city and community authorities. These authorities granted permission to operate in the community and also to make use of designated places such as community halls or nearby schools for data and sample collection.

Bellville South is located within the northern suburbs of Cape Town, South Africa. It is traditionally a mixed-ancestry township formed in the late 1950s. In the South African context, the term township usually refers to the often underdeveloped urban living areas that, under the Apartheid regime, were reserved for people of mixed ancestry. According to the 2001 population census, its population was approximately 26 758, with the people of mixed ancestry making up 80.48% (21 536). The target population for this study were subjects between the ages of 35 and 65 years and their number was estimated to be 6 500.^21^ Based on these statistics and the recommended sample size for a pilot study, usually 10%, the sample size required for this pilot study was 650.

This was a cross-sectional study aimed at establishing a cohort that could be followed up for insulin resistance and its sequel in randomly selected mixed-ancestry subjects aged 35 to 65 years. The data presented here were collected from mid January 2008 to March 2009. Using a map of Bellville South, random sampling was approached as follows. From a list of streets from each stratum, the streets were then classified as short, medium and long streets based on the number of houses. Streets with 22 or fewer houses were classified as short, streets with 23–40 houses were medium, and long streets were those with more than 40 houses. A total of 16 short streets representing approximately 190 houses, 15 medium streets representing approximately 410 houses and 12 long streets representing approximately 400 houses were randomly selected across the different strata.

From the selected streets, all household members meeting the selection criteria were eligible to participate in the study. Community authorities requested that participants outside the random selection area should benefit from the study. These were also included, but given a different code.

Information regarding the project was disseminated to the residents through the local radio station (Radio Tygerberg) and community newspaper (the Tygerberger). Brochures and fliers bearing information about the project were distributed via the school children and taxis to the local residents. The recruitment team consisted of unemployed matriculants and was managed by a qualified, retired nurse from the community. Additionally, a ‘road show’ strategy that involved a celebrity suffering from diabetes from the same community was also used, especially in the targeted streets.

Recruited subjects were visited by the recruitment team the evening before participation and reminded of all the survey instructions. The instructions included overnight fasting, and abstinence from drinking alcohol or consumption of any fluids on the morning of participation. Since the participants were required to bring in an early morning mid-stream urine sample, they were provided with a sterile container as well as instructions on how to collect the sample. Furthermore, participants were encouraged to bring along their medical/clinic cards and/or drugs they were currently using.

## Data collection

A detailed protocol describing data-collection procedures (questionnaires and physical examination) was developed. The team members, consisting of professional nurses and the recruitment team, were trained and a pilot study in a neighbouring community with similar demographics was performed to validate the questionnaire and to synergise the workflow. A supervisor, who monitored the performance of the personnel and who was responsible for calibrating equipment according to a standard protocol, was allocated to each team. In addition, a weekly meeting was held to assess progress, solve problems and re-train the research team.

A questionnaire designed to retrospectively obtain information on lifestyle factors such as smoking and alcohol consumption, physical activity, diet, family history of CVD and DM, and demographics was administered by trained personnel. The questionnaire was adapted from several existing standard and recognised sources,^22^, ^23^ and was also pre-tested in a neighbouring community with similar demographics. A detailed drug history was obtained by interrogation and by examining the clinic cards as well as the record of drugs that participants brought to the study site.

Clinical measurements included height, weight, hip and waist circumferences, body fat measurements (subscapular and supra-iliac skin-fold thickness, triceps and mid upper-arm/biceps circumference) and blood pressure. Qualified healthcare professionals who underwent training to standardise all measurements prior to the commencement of the study carried out measurements.

Blood pressure measurements were performed according to WHO guidelines.^24^ Measurements were performed using a semi-automatic digital blood pressure monitor (Rossmax PA, USA) on the right arm in a sitting and ambulatory position. After a 10-minute rest period, three readings were taken at five-minute intervals and the lowest of the three readings was taken as the blood pressure.

Weight was determined on a Sunbeam EB710 digital bathroom scale, which was calibrated and standardised using a weight of known mass. Weight measurements were recorded to the nearest 0.1 kg and taken with each subject wearing light clothing, without shoes and socks. Height was recorded in centimetres to one decimal place using a stadiometer, with subjects standing on a flat surface at a right angle to the vertical board of the stadiometer. Body mass index (BMI) was calculated as weight per square metre (kg/m^2^).

Waist circumference was measured using a non-elastic tape at the level of the narrowest part of the torso, as seen from the anterior view. If it was difficult to see the waist narrowing, especially in obese subjects, the waist circumference was measured between the ribs and the iliac crest. All anthropometric measurements were performed three times and the average measurements were used for analysis.

All participants except the self-reported diabetic subjects, confirmed by either medical card record or drugs in use, underwent a 75-g oral glucose-tolerance test (OGTT) as prescribed by the WHO, with fasting blood glucose determinations in all participants. Categories of glucose tolerance were defined applying the 1998 WHO criteria.^25^ Blood samples were transported daily in an icebox for processing at the Metropolis private pathology laboratory (Century City, Cape Town).

## Analyses

Plasma glucose was measured by the enzymatic hexokinase method (Cobas 6000, Roche Diagnostics). Glycosylated haemoglobin (HbA_1c_) was assessed by turbidimetric inhibition immunoassay (Cobas 6000, Roche Diagnostics). High-density lipoprotein cholesterol (HDL-C) and triglycerides (TG) were estimated by enzymatic colorimetric methods (Cobas 6000, Roche Diagnostics). Low-density lipoprotein cholesterol (LDL-C) was calculated using Friedwald’s formula.

Gender-specific prediction for CVD risk was calculated using the 30-year CVD interactive risk calculator.^16^ The calculator uses standard CVD risk factors (male gender, age, systolic blood pressure, antihypertensive treatment, diabetes mellitus, total and HDL-C or BMI instead of lipids to predict two outcomes: hard CVD (coronary death, myocardial infarction, fatal or non-fatal stroke) and full CVD (hard CVD or coronary insufficiency, angina pectoris, transient ischaemic attack, intermittent claudication or congestive heart failure).

## Statistical analysis

Statistical analysis of the data was performed using STATISTICA (STATISTICA 9, StatSoft Inc 1984–2009). The continuous variables are presented as median (25th, 75th quartile range) for asymmetrical data or means ± standard deviation (SD) for normally distributed data, and categorical variables are expressed in percentages. For data where the normality assumptions were suspect, the Mann Whitney U-test was used. The Chi-square test was used for comparison of categorical variables.

Analysis of covariance, with age as covariate, was used for the comparison of continuous variables between the subjects with IFG, IGT, newly diagnosed diabetes, self-reported diabetes and the control group with normal glucose tolerance. Factorial ANOVA was used to compare the CVD risk in normal-weight and obese subjects in the diabetic and non-diabetic subjects. Best-subset linear regression analysis was done with estimated 30-year Framingham risk as dependent variable. The lipid- and BMI-dependant equations were compared using intra-class correlation (ICC) calculated by the R programming language.

## Results

A total of 956 subjects participated, comprising 642 random subjects between the ages of 35 and 65 years and 304 voluntary subjects, age range 16–95 years. The other 10 subjects were from other race groups and were excluded. For this study, 600 subjects were between the ages of 20 and 60 years, 13 were excluded because they were already on treatment for CVD, three did not consent to giving blood samples, while one had no blood pressure measurements, resulting in a total of 583 subjects.

Table 1 presents the general characteristics of the 583 participants eligible for this study, and data are presented as medians (25th, 75th quartile range). Although the BMI and waist circumference of females were significantly elevated over that of males (*p* < 0.0001), the CVD risk was significantly higher in males (*p* < 0.0001).

**Table 1:** Characteristics Of Cohort, Stratified By Gender

*Characteristics*	*Male (n = *126*)*	*Female (n = *457*)*	p
Age (years)	47 (40, 55)	46 (39, 53)	0.31
BMI (kg/m^2^)	25.0 (20.8, 29.0)	30.3 (25.9, 35.1)	< 0.0001
WC (cm)*	90.3 (77.8, 100.8)	97.0 (86.5, 108)	< 0.0001
Hip C (cm)*	98 (91, 99)	112 (103, 112)	< 0.0001
SBP (mmHg) *	121 (113, 130)	117 (106, 129)	0.0065
DBP (mmHg)*	76 (70, 85)	74 (67, 83)	0.0372
FBG (mmol/l)	5.3 (4.7, 6.1)	5.4 (5.0, 6.1)	0.2717
PostBG (mmol/l)	6.0 (5.1, 7.9)	6.6 (5.6, 8.2)	0.0068
HbA_1c_ (%)	5.7 (5.5, 6.2)	5.7 (5.4, 6.2)	0.7793
TC (mmol/l)	5.2 (4.4, 5.9)	5.5 (4.7, 6.3)	0.0200
TG (mmol/l)	1.29 (0.91, 1.78)	1.19 (0.85, 1.71)	0.2462
HDL-C (mmol/l)	1.13 (0.95, 1.41)	1.21 (1.01, 1.44)	0.0300
LDL-C (mmol/l)	3.23 (2.54, 3.9)	3.56 (2.94, 4.2)	0.0011
Lipid full (%)	41.5 (24, 66)	31 (16, 51)	< 0.0001
Lipid hard (%)	29.5 (15, 52)	17 (8, 32)	< 0.0001
BMI full (%)	46 (27, 61)	32 (18, 53)	< 0.0001
BMI hard (%)	32 (17, 55)	18 (9, 33)	< 0.0001

*Replicated measurements; BMI, body mass index; WC, waist circumference; Hip C, hip circumference; SBP, systolic blood pressure; DBP, diastolic blood pressure; FBG, fasting blood glucose; PostBG, post 2-hour blood glucose; TC, total cholesterol; TG, triglyceride; HDL-C, high-density lipoprotein cholesterol; LDL-C, low-density lipoprotein cholesterol.

**Fig. 1:**
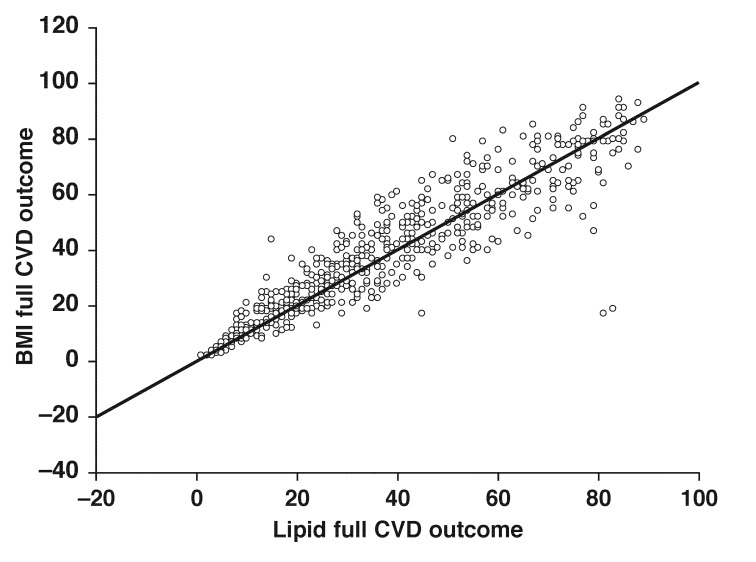
Intra-class correlation between BMI and lipiddependent equation for full CVD outcome. Intra-class correlation agreement = 0.920, standard error of measurement 6.4%.

In this study, both lipid- and BMI-dependant equations were used, and the intra-class correlation (ICC) between the two equations was 0.92 with a 6% standard error of measurement (SEM) (Fig. 1). The results are therefore presented using the lipid-dependent equation.

**Fig. 2:**
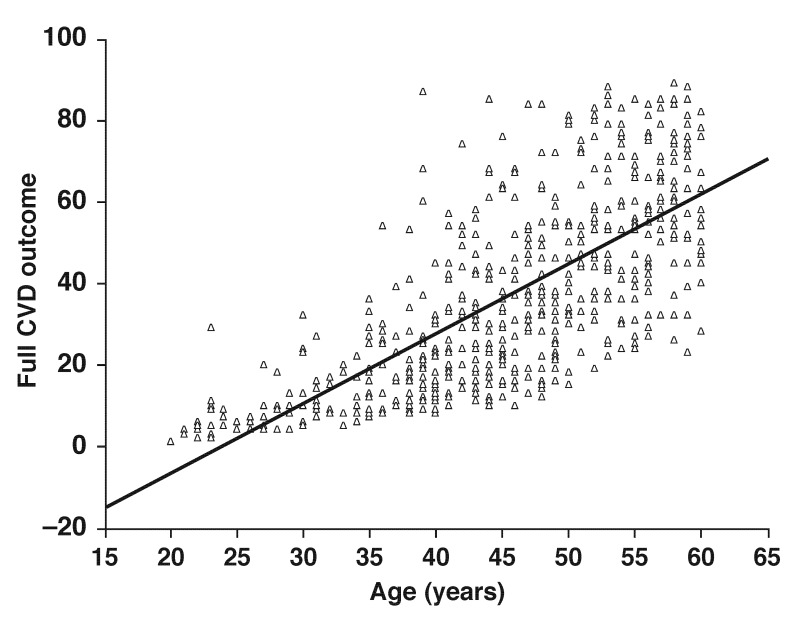
CVD risk score for men and women in relation to their age.

The pattern of CVD risk factors used in the 30-year risk calculator is shown in Table 2. Generally, diabetes, hypertension and percentage CVD risk increased with age. On the other hand smoking was more prevalent in the younger age group, 20 to 30 years, while overweight (BMI ≥ 25, < 30 kg/m^2^) was similar across age groups. While diabetes was highest in the age group 51 to 60 years, undiagnosed diabetes was present in all age groups. The scatter plot in Fig. 2 illustrates the effect of age on increasing CVD risk. Even in those subjects younger than 35 years, some had CVD risk of 20% or more.

**Table 2:** Cvd Risk Factors Used In The Equation In Different Age Groups

*Age groups (years)*				
*CVD risk factor*	*20–30*	*31–40*	*41–50*	*51–60*
% males	9.5	16.7	36.50	37.3
% BMI < 25 kg/m^2^	50	22.95	30.81	20.71
% BMI ≥ 25, < 30 kg/m^2^	29.17	24.59	25.12	29.80
% BMI ≥ 30 kg/m^2^	20.83	52.46	44.08	49.49
SBP (mmHg)*	112.7 ± 13.9	113.9 ± 13.7	119.4 ± 17.1	125.6 ± 17.6
TC (mmol/l)*	4.7 ± 1.1	5.1 ± 1.0	5.4 ± 1.1	6.0 ± 1.2
HDL-C (mmol/l)*	1.2 ± 0.26	1.2 ± 0.37	1.3 ± 0.35	1.3 ± 0.36
% smoking	63.27	49.18	47.42	44.72
TRTBP	10.42	12.30	25.59	47.45
*Diabetes status*				
% IFG	2.04	4.96	5.63	3.52
% IGT	2.04	13.22	15.02	20.60
% undiagnosed DM	4.08	5.79	13.62	18.59
% self-reported DM	0	5.79	6.57	14.07
Lipid full (%)^#^	8.5 ± 6.9	19.4 ± 13.0	35.1 ± 17.6	56.6 ± 18.2

*Replicated measurements, ^#^Mean ± standard deviation. BMI, body mass index; SBP, systolic blood pressure; TC, total cholesterol; HDL-C, high-density lipoprotein cholesterol; TRTBP, treatment for blood pressure; IFG, Impaired fasting glucose; IGT, impaired glucose tolerance, DM, diabetes mellitus.

**Fig. 3:**
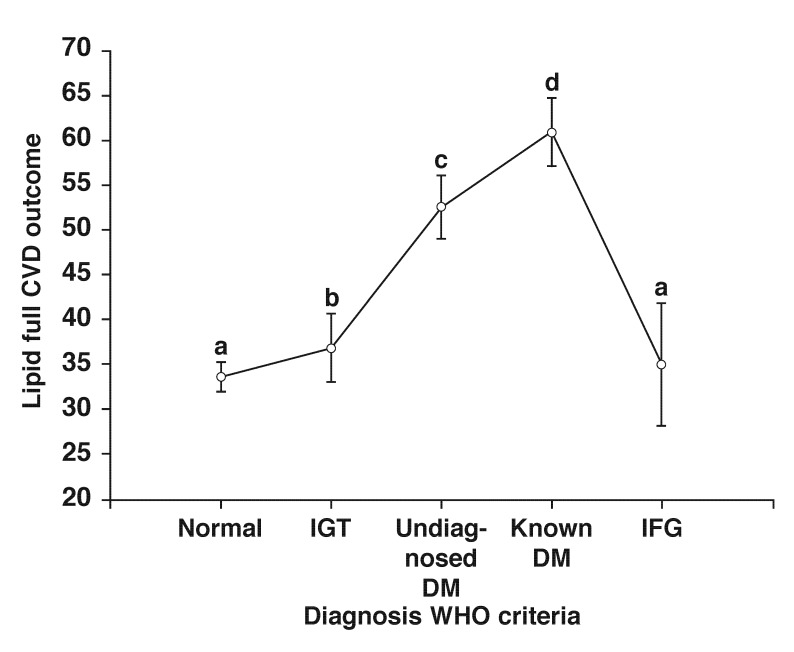
Covariance analysis with age as covariate between the subjects with IFG, IGT, newly diagnosed diabetes (undiagnosed DM), self-reported diabetes (known DM) and subjects with normal glucose tolerance (normal). Significant differences between the groups are denoted by the letters, a–d. No significant differences were observed between those with IFG and normal glucose tolerance. Vertical bars denote 0.95 confidence interval.

The mean 30-year CVD risk among individuals without diabetes was 33.6%, while in those with undiagnosed and self-reported diabetes it was more than 50%, (*p* < 0.001) (Fig. 3). In normoglycaemic females who were of normal weight (BMI < 25 kg/m^2^), the CVD risk was significantly lower (*p* = 0.007) than in obese females, while in hyperglycaemic states, observed differences were not significant (Table 3). In the best-subsets linear regression analysis, the significant predictors of CVD were sibling history of diabetes, and TG, LDL-C and HbA_1c_ levels (*p* < 0.001) (Table 4). These variables accounted for 46.3% of the variation in the calculated CVD risk.

**Table 3:** Cvd Risk Stratified By Bmi And Glycaemic State

	*Females*		*Male*	
	*Non-diabetic*	*Diabetic*	*Non-diabetic*	*Diabetic*
BMI < 25 kg/m^2^	20.8 (17.7–23.9)	49.7 (33.5–65.9)	27.6 (22.4–32.9)	55.1 (42.5–67.7)
BMI ≥ 25 < 30 kg/m^2^	24.8 (20.4–29.1)	65 (57.8–72.2)	43.5 (32.6–54.4)	72 (64.3–79.7)
BMI ≥ 30 kg/m^2^	31.3 (27.9–34.7)**	57.8 (52.3–63.3)	42.3 (28.3–56.3)	71.9 (60.8–83.0)

Mean (95% CI). Diabetic refers to all diabetic subjects including those diagnosed during the survey; non-diabetic excludes those with IGT or IFG. **Significant difference between non-diabetic female BMI < 25 kg/m^2^ and non-diabetic female BMI ≥ 30 kg/m2, (*p* = 0.007).

**Table 4:** Best-Subset Linear Regression Analysis. Adjusted R^2^ = 0.46

*Variable*	b*	b	p
Sibling history of DM	0.14	9.16	< 0.0001
TG (mmol/l)	0.34	6.67	< 0.0001
LDL-C (mmol/l)	0.33	7.24	< 0.0001
HbA_1c_ (%)	0.34	4.86	< 0.0001

Other variables initially included in the regression analysis were waist circumference, subscapular and supra-iliac skin thickness, biceps and triceps circumference, brother, sister, mother or father history of diabetes and/or hypertension, death of parent from CVD, C-reactive protein, and HOMA IR. TG, triglycerides; LDL, low-density lipoprotein cholesterol.

## Discussion

In this study, the CVD risk was significantly higher in subjects with diabetes. It is well documented that individuals with type 2 DM have increased CVD risk compared to those without diabetes, and the 30-year risk among subjects with diabetes has been shown to proportionally increase with BMI.^26^, ^27^ However, no significant differences were observed between obese and normal-weight diabetics in this study.

In the Framingham cohort, the lifetime risk in obese diabetic subjects was 78.8 and 86.9% in women and men, respectively.^27^ Furthermore, approximately half of those with diabetes were unaware that they had the disease. Chronic hyperglycaemia, even in the absence of symptoms, is associated with an increased risk of diabetic micro-angiopathy and CVD.^7^ Undiagnosed hyperglycaemia has previously been demonstrated in rural and urban South Africans^2^, ^3^ and a recent study found the presence of undiagnosed hyperglycaemia in patients with coronary artery disease.^28^

While significant differences were observed between the estimated CVD risk in non-diabetic (excluding IFG and IGT) normal-weight and obese subjects, particularly in females, high risk scores were still evident in the normal-weight, normoglycaemic individuals. The present study builds on a previous study that found the 10-year CVD risk profile in a similar population to be less than 10% in subjects under 35 years of age.^29^

Ten-year risk estimates have been criticised because they underestimate the risks, allowing for continued progression of sub-clinical atherosclerosis. Indeed this is evident in our study in which we present evidence of a high risk score (> 20%) in young individuals and in those subjects with normoglycaemia (Fig 2) and (Fig 3). Similarly, younger individuals of the Framingham cohort with very low 10-year CHD risk were shown to still have a substantial lifetime risk of CHD.^30^ Overall, these data confirm that irrespective of glycaemic or weight status, an evidence-based tool is crucial for the identification of high-risk subjects.

Given the findings of this study and the estimated increases in the incidence of both diabetes and hypertension in South Africa, this trend may continue to worsen if current trajectories do not change. Hypertension, a condition that was rarely observed in non-Western populations in the 1940s, has emerged as the most common cause of heart failure in Africa.^31^, ^32^ This has been attributed to the adoption of Western lifestyle and diet, with a parallel increase in obesity, diabetes and hypertension.^32^ Evidence from systematic studies suggests that CVD risks that were almost unprecedented in non-Caucasian South Africans are now apparent in both rural and urban adult populations.^33^-^37^

The intra-class correlation between the lipid- and BMI-based equations showed that either method could be used for the estimation of CVD risk. In developing countries, the use of methods that do not require blood or invasive testing or those that can use information that is easily obtainable in a primary healthcare setting has been advocated. Except for confirmation of the diabetes status, all measurements required to estimate an individual’s CVD risk can be obtained in such a setting. Point-of-care instruments are usually available in these healthcare facilities to screen for diabetes and to assess one’s blood glucose levels and could adequately suffice for the purpose of CVD estimation. Normally, those with high glucose levels or equivocal results have blood drawn for confirmation. Unfortunately, the subset linear regression analysis showed that except for one, the other risk factors that are associated with a high CVD risk also require blood testing.

Our study observed a strong association between HbA_1c_ and the estimated CVD risk. Clinical trials have convincingly demonstrated that good glycaemic control can reduce the risk of CVD in patients with both type 1 and type 2 diabetes.^38^-^40^ However, it has recently been indicated that accelerated atherosclerosis and CVD in diabetes is likely to be multifactorial and therefore a glucocentric approach is discouraged in the management of diabetes on cardiovascular outcomes.^41^, ^42^ On the other hand, the strong association of triglycerides and LDL-C suggests that in some asymptomatic individuals, lipid-lowering therapy may have to be initiated.

The major limitation of this study was the use of a risk calculator that was developed in a predominantly white middle-income cohort. The mixed ancestry, sometimes referred to as coloured, is a South African population group with Khoi, San, Malay, European and African ancestry,^29^ and is relatively poor. However, the INTERHEART and Heart of Soweto studies have shown that the common risk factors for heart diseases in Africans resemble those observed in Western societies.^19^, ^37^ Until such time as a validated and recalibrated equation has been developed, this predictive risk calculator may provide a rough estimate of CVD risk in the absence of any reliable tool.

As indicated, approximately a third of the study group (mainly those under 35 years) were self-selected. However given the relatively low incidence and ignorance of CVD risk, particularly in this age group, it is unlikely to have biased the findings of this study.

## Conclusion

Although the estimated CVD risk was highest in those with hyperglycaemia, it was also evident in normal-weight, normoglycaemic and younger individuals. Results from this pilot study have important public health implications as CVD is often underestimated in the young. A significant number of participants were unaware of their diabetic status, pointing to the need to identify this group as a potentially high-risk CVD group.

Due to inter-convertibility of lipids and BMI in the risk-estimation equation, CVD risk can easily be estimated in a primary healthcare setting. In South Africa, there has been mounting pressure for the reorganisation of the primary healthcare system to develop an approach for the management of chronic diseases. In addition to improving the primary healthcare system, another approach would be the inclusion of CVD education in school and university curricula, as is currently being done for HIV/AIDS. This may also assist in reducing the prevalence of obesity and the metabolic syndrome, which we recently reported in South African children.^43^
